# Benchmark of filter methods for feature selection in high-dimensional gene expression survival data

**DOI:** 10.1093/bib/bbab354

**Published:** 2021-09-08

**Authors:** Andrea Bommert, Thomas Welchowski, Matthias Schmid, Jörg Rahnenführer

**Affiliations:** Department of Statistics, TU Dortmund University, Vogelpothsweg 87, 44227, Dortmund, Germany; Institute of Medical Biometry, Informatics and Epidemiology (IMBIE), Medical Faculty, University of Bonn, Venusberg-Campus 1, 53127, Bonn, Germany; Institute of Medical Biometry, Informatics and Epidemiology (IMBIE), Medical Faculty, University of Bonn, Venusberg-Campus 1, 53127, Bonn, Germany; Department of Statistics, TU Dortmund University, Vogelpothsweg 87, 44227, Dortmund, Germany

**Keywords:** benchmark, feature selection, filter methods, high-dimensional data, survival analysis

## Abstract

Feature selection is crucial for the analysis of high-dimensional data, but benchmark studies for data with a survival outcome are rare. We compare 14 filter methods for feature selection based on 11 high-dimensional gene expression survival data sets. The aim is to provide guidance on the choice of filter methods for other researchers and practitioners. We analyze the accuracy of predictive models that employ the features selected by the filter methods. Also, we consider the run time, the number of selected features for fitting models with high predictive accuracy as well as the feature selection stability. We conclude that the simple variance filter outperforms all other considered filter methods. This filter selects the features with the largest variance and does not take into account the survival outcome. Also, we identify the correlation-adjusted regression scores filter as a more elaborate alternative that allows fitting models with similar predictive accuracy. Additionally, we investigate the filter methods based on feature rankings, finding groups of similar filters.

## 1 Introduction

Feature selection is one of the most fundamental problems in the analysis of high-dimensional data. Especially for high-dimensional data sets, it is often advantageous with respect to predictive performance, run time and interpretability to disregard the irrelevant and redundant features. This can be achieved by choosing a suitable subset of features that are relevant for target prediction. In bioinformatics, feature selection often allows identifying the features that are important for biological processes of interest. Due to the enormous amount of existing feature selection methods, benchmark studies are of great importance for identifying the best methods to use in data analyses.

In the past decades, many feature selection methods have been proposed. The methods can be categorized into three classes: filter methods, wrapper methods and embedded methods [1]. Filter methods rank features by calculating a score for each feature independent of a model. Either the $l$ features with the highest scores or all features whose scores exceed a threshold $\tau $ are selected (with $l \in \mathbb{N}$ or $\tau \in \mathbb{R}$ being pre-specified). For many filter methods, the score calculation can be done in parallel, thus resulting in increased computational efficiency. An extensive overview of existing filter methods is given in [2]. Wrapper methods [3] consider subsets of the set of all features. For each of the subsets, a predictive model is fitted and the subsets are evaluated by a performance measure calculated from the resulting model. Wrapper methods include not only simple approaches like greedy sequential searches [4], but also more elaborate algorithms like recursive feature elimination [5] as well as evolutionary and swarm intelligence algorithms for feature selection [6–8]. Embedded methods include the feature selection in the model fitting process. Examples of predictive methods that perform embedded feature selection are lasso regression [9] and tree-based methods like random forests [10] or gradient boosting [11, 12]. There are many overview papers that describe in detail, categorize and suggest how to evaluate existing feature selection methods, for example [1, 13–21].

Regarding the comparison of different methods, benchmark studies have gained increasing attention. The majority of these studies are based on classification data. For these, the feature selection methods are combined with classification methods in order to assess the predictive performance of the selected features. In [22], filter methods are compared based on two gene expression data sets, counting the number of misclassified samples. In [23], the classification accuracy of different filter, wrapper, and embedded methods on several artificial data sets is analyzed. In [24, 25], filter methods are compared with respect to classification accuracy based on microarray data sets. In [26, 27], extensive comparisons based on text classification data sets are conducted. The authors analyze filter and wrapper methods, respectively. In [28], filter methods are compared with respect to classification accuracy on malware detection data. In [29, 30], filter methods are studied on large data sets, analyzing the predictive accuracy with respect to the number of features to select. In [31, 32], small artificial data sets are used to assess whether the correct features are selected. In [31], different feature selection methods are compared while in [32], only filter methods are considered. In [33], filter and wrapper methods are compared on large simulated data sets with respect to the correctness of the selected features. Additionally, the authors conduct comparisons with respect to classification accuracy on real data sets. In [34], filter and wrapper methods are compared comprehensively with respect to classification accuracy and run time, considering each of the two objectives separately. Most of the data sets on which the comparison is based contain a small or medium number of features. In [35, 36], several filter methods that are based on mutual information are compared. In [35], the accuracy and the run time of the methods are analyzed separately. Additionally, the authors take into account theoretical properties and look at the proportions of correctly identified features on artificial data. In [36], the authors analyze the classification accuracy with respect to the number of selected features and search for Pareto optimal methods considering the accuracy and feature selection stability. In [37], an extensive study of correlation-based feature selection is conducted. The author analyzes the classification accuracy based on real data sets as well as the choice of relevant or irrelevant features based on artificial data sets. In [38], an extensive comparison of 22 filter methods on 16 large or high-dimensional data sets is conducted with respect to both classification accuracy and run time jointly. Also, the empirical similarity of the filter methods based on the rankings of all features of all considered data sets is analyzed. In [39], the authors perform hyper parameter tuning of predictive models on survival data sets. They use combined methods consisting of a filter and a survival prediction method and consider the choice of filter method as a hyper parameter. This way, they find out which filter methods yield good results on many data sets.

In this article, we analyze feature selection methods on gene expression survival data sets. The features of gene expression data contain information about the activity of genes, for example in cancerous tissues. Gene expression data are a typical example of high-dimensional data used in bioinformatics. The outcome of survival data consists of two variables: one variable indicating the observed outcome time and one variable indicating if an event occurred or if the observation is censored. For right-censored data, a censored observation means that no event has occurred up to this point in time, but no information about events occurring afterward is available. For survival data sets, commonly used regression or classification techniques are not suitable because they cannot take into account the censoring information. Since censored survival data are frequently used in bioinformatics and since feature selection methods for survival data have not been thoroughly benchmarked before, it is necessary to conduct such a benchmark for survival data.

This article focuses on the comparison of filter methods for feature selection. Our focus on filter methods is motivated by the following considerations: Most wrapper methods are computationally infeasible for high-dimensional data sets. Embedded methods require the use of a certain predictive model. Most filter methods, however, are fast to calculate and can be combined with any kind of predictive method, even methods with embedded feature selection, see [40, 41]. Also, they can heavily reduce the run time for fitting the subsequent model. So, for data sets with really large numbers of features, it can be necessary to pre-filter the data set in order to make further analyses possible. To the best of our knowledge, a thorough and extensive filter comparison study has not yet been conducted for survival data.

In this article, 14 filter methods from different toolboxes are benchmarked based on 11 gene expression survival data sets. The filter methods considered here are representatives of the most prominent general concepts for filter methods. These classes of filter methods are univariate filters, feature importance filters based on multivariate models and information theoretic measures. Most of the compared filter methods have been integrated into the machine learning *R* package *mlr3* [42] and are ready to use. *mlr3* is a comprehensive package for machine learning and a standard in the *R* community.

The aim of benchmarking the filter methods is to identify the best filter methods, so that these methods can be employed in future data analyses. The best filter methods are assessed with respect to predictive performance when combined with a predictive model and with respect to run time. Also, the number of selected features required for obtaining a model with high predictive accuracy and the feature selection stability is assessed. Feature selection stability is defined as the robustness of the set of selected features with respect to different data sets from the same data generating distribution and is crucial for the reliability of the results [43]. Additionally, we analyze the empirical similarity of the filter methods. For finding groups of similar filter methods, we investigate which methods select the top features in a similar order. Our analysis identifies three groups of filter methods with a similar behavior, as well as several filter methods that are not very similar to any other filter method. There is one simple filter method that performs best with respect to predictive accuracy, run time and feature selection stability. We also identify a more elaborate filter method that allows fitting models with similar predictive performance.

The remainder of this article is organized as follows: In the Methods section, basic concepts of survival data are briefly introduced, the filter methods considered in this article are described and a feature selection stability measure is defined. In the Experiments section, the considered data sets are given. For both the similarity analysis and the comparison of the filters’ performances, the setup of the experiments is explained and the results are analyzed in detail. The Conclusions section contains a summary of the findings and concluding remarks.

## 2 Methods

### 2.1 Survival data basics

#### 2.1.1 Notation

A data set with $n$ observations of the $p$ features $X_1, \ldots , X_p$ is considered. Furthermore, $T$ denotes the observed outcome time and is defined as $T = T_{\textrm{obs}} = \min \{T_{\textrm{true}}, C\}$ with $T_{\textrm{true}}$ denoting the true survival time and $C$ denoting the censoring time. $\Delta = \mathbb{I}(T_{\textrm{true}} \leq C)$ is the event indicator with ‘0’ corresponding to a right-censored and ‘1’ corresponding to an uncensored observation.

#### 2.1.2 Cox proportional hazards regression model

The Cox proportional hazards model is a regression technique for modeling survival outcomes. It is defined as

(1) \begin{align*}& h(t,x) = h_0(t) \cdot \exp(x^\intercal \beta) \end{align*}

with $h(t,x)$ denoting the hazard at time $t$ for an individual with covariate vector $x$ representing the features. The term $h_0(t)$ denotes the baseline hazard at time $t$ and $\beta $ is the vector of regression parameters. When fitting a Cox proportional hazards model to a data set, the baseline hazard and the regression parameters are estimated by maximizing the partial log-likelihood. For more details about Cox proportional hazards models see [44].

A variant of the Cox proportional hazards model is the $L_2$-regularized Cox proportional hazards model [45]. For the parameter estimation of this model, the partial log-likelihood is modified with a $L_2$-penalty. The $L_2$-penalty shrinks all regression coefficients toward zero. In contrast to an unregularized Cox proportional hazards model, a $L_2$-regularized Cox proportional hazards model can be fitted on data sets that contain more features than observations.

#### 2.1.3 Integrated Brier score

The integrated Brier score [46] is a measure of prediction error that reflects both discrimination and calibration aspects. It is estimated using inverse probability weighting. The integrated Brier score measures the quadratic difference between the individual estimated survival function of each observation and its observed indicator function of individual survival and then sums these values for all observations. The range of the integrated Brier score is $[0,1]$ with 0 being the best value.

#### 2.1.4 Transforming survival data into regression data using martingale residuals

Some of the filter methods described below require the transformation of the survival outcome to an uncensored continuous outcome. This can be accomplished by calculating martingale residuals [47]. For a survival data set with right-censored data, first, a Cox proportional hazards regression model is fitted without covariates. This model incorporates the information given by the observed outcome time $T$ and the event indicator $\Delta $. Then, for each observation, the martingale residual is calculated based on the Cox model. Martingale residuals are real-valued and can be used as uncensored continuous outcomes. For more details see [47]. In the following subsection, we will refer to the transformed target variable containing the martingale residuals as $Y^{(m)}$.

### 2.2 Filter methods

In this subsection, the 14 filter methods analyzed in this article are defined, using the notation of the previous subsection. All filter methods in this subsection are described for survival data sets with continuous features, because gene expression data sets usually only contain continuous features. Two kinds of filter methods are presented: Most filter methods calculate a score for all features and then select the features with the highest scores. Some filter methods, however, select features iteratively in a greedy forward fashion. For these filters, in each iteration, the feature with the maximal score is selected but the scores of different iterations are not comparable. We first describe univariate filter methods, which do not consider interactions between the $p$ features. Subsequently, we discuss multivariate filter methods. All filter methods are described in the way they are implemented in the software used for the comparative experiments, which is indicated at the end of this subsection.

#### 2.2.1 Univariate filter methods

The *variance* filter uses the variance of a feature as its score

(2) \begin{align*}& S_{\textrm{variance}}(X_k) = \textrm{variance}\ (X_k). \end{align*}

The idea of this filter is to remove features that only consist of noise and therefore have very little variation. This filter only makes sense for data sets where the features are measured on the same scale and have not been scaled to unit variance.

For the *correlation* filter, the survival targets are transformed into the continuous target variable $Y^{(m)}$ as described above. Then, for each feature, the absolute Pearson correlation between this feature and $Y^{(m)}$ is computed and used as filter score

(3) \begin{align*}& S_{\textrm{correlation}}\left(X_k, Y^{(m)}\right) = \left| \textrm{Cor}\left(X_k, Y^{(m)}\right) \right|. \end{align*}

The idea of this filter is to keep features that have a strong linear association with the target variable.

The *cox.score* filter assesses how well each feature can explain the survival outcome univariately. For feature $X_k$, a Cox proportional hazards regression model with only $X_k$ as independent variable is fitted. Then, a score test [44] is calculated for this model. The test statistic of the score test is used as filter score

(4) \begin{align*}& S_{\text{cox.score}}(X_k, T, \Delta) = \textrm{test statistic of score test for} X_k. \end{align*}

The idea of this filter is that, the more important a feature is for explaining the survival outcome, the higher is the test statistic of the respective score test.

The *carss* filter calculates absolute correlation-adjusted regression survival scores $S_{\textrm{carss}}(\boldsymbol{X}, \log (T), \Delta )$, in which $\boldsymbol{X} \in \mathbb{R}^{n \times p}$ is defined as the matrix containing all covariates and $\log (T)$ corresponds to the logarithmic observed outcome time. To estimate these scores, first, the correlation between $\log (T)$ and each covariate $X_k \in \boldsymbol{X}$ is adjusted by inverse probability of censoring weighting [48] in the formula

(5) \begin{align*}& \boldsymbol{R}_{\boldsymbol{X}, \log(T)} = \left( \frac{S_{X_k,\log(T);w}} {\sqrt{S_{X_k}^2} \sqrt{S_{\log(T);w}^2}} \right)_{k = 1, \dots, p}. \end{align*}

This equation yields the usual correlation definition with the weighted covariance $S_{X_k,\log (T);w}$, weighted variance $S_{\log (T);w}^2$ and variance $S_{X_k}^2$. The weights $w$ are calculated based on an estimate of the Kaplan–Meier survival function of the logarithmic censoring process. Then, the estimated correlations $\boldsymbol{R}_{\boldsymbol{X}, \log (T)} \in \mathbb{R}^{p \times 1}$ are decorrelated with the estimated covariate correlation matrix $\boldsymbol{R} \in \mathbb{R}^{p \times p}$ by

(6) \begin{align*}& S_{\textrm{carss}}(\boldsymbol{X}, \log(T), \Delta) = \left| \boldsymbol{R}_{\textrm{shrink}}^{-1/2} \, \boldsymbol{R}_{\boldsymbol{X}, \log(T)} \right|. \end{align*}

The matrix $\boldsymbol{R}_{\textrm{shrink}}^{-1/2}$ is estimated by shrinkage toward the identity matrix following the approach of [49, 50]. No additional tuning parameter is necessary, because the amount of shrinkage can be estimated directly from the data [51]. For a more detailed description of this method, we refer to [52].

#### 2.2.2 Filter methods based on feature importance measures

Three feature importance filters based on multivariate models are considered: random forest permutation importance, random forest impurity importance and gradient boosting importance. Random survival forests are bagging ensembles with survival trees as base learners [10]. To calculate the permutation importance, the out of bag (oob) observations for each tree, that is, the observations that were not used for fitting this tree, are considered. For the oob observations of each tree, feature $X_k$ is permuted. Then, for the permuted observations, the cumulative hazards are predicted by the corresponding trees. The resulting predictive accuracy is compared to the predictive accuracy without permuting feature $X_k$. To measure the predictive accuracy, the concordance index (C-index) by Harrell [53] is employed, see [10] for details on computing the C-index based on cumulative hazards. The score of the *permutation* importance filter is the decrease in predictive accuracy of the random forest from original oob observations to permuted observations. Features that are important for survival prediction cause a large decrease in accuracy because their relevant information is not available when the feature is permuted.

(7) \begin{align*} &S_{\textrm{permutation}}(X_k, T, \Delta) \nonumber \\ & = \textrm{random forest permutation importance of}\ X_k. \end{align*}

The *impurity* filter considers the node impurities of the trees. Nodes containing observations with similar survival are called pure, nodes with many dissimilar cases are called impure. When constructing a survival tree, the split variables and split points are chosen based on maximal difference in survival measured by the statistic of the log-rank test. To assess the gains in purity due to feature $X_k$, the sum of all log-rank test statistics for all splits based on $X_k$ is calculated and used as filter score. A feature that is important for survival prediction causes on average a large gain in purity.

(8) \begin{align*} &S_{\textrm{impurity}}(X_k, T, \Delta) \nonumber \\ & = \textrm{random forest impurity importance of}\ X_k. \end{align*}

The *boosting* filter uses the gradient boosting feature importance as filter score. Gradient boosting is an ensemble method that additively combines many weak learners into one strong prediction method [11, 12]. Here, survival trees (actual trees, not just stumps) are used as weak learners and the negative partial log-likelihood is employed as loss function. To assess the importance of the features in the boosting model, first, for each split in each tree, the improvement caused by this split is assessed. Then, for each feature, the sum of the corresponding improvement values is calculated [54]. A feature that is important for survival prediction causes on average large improvements.

(9) \begin{align*}& S_{\textrm{boosting}}(X_k, T, \Delta) = \textrm{gradient boosting importance of}\ X_k. \end{align*}

#### 2.2.3 Mutual information-based filter methods

For applying the following mutual information-based filter methods, a data set with categorical features and categorical target variable is required. To transform a survival data set accordingly, first, the continuous variable $Y^{(m)}$ is created as described above. Then, $Y^{(m)}$ is transformed into the categorical target variable $Y^{(c)}$ by cutting its range into $q$ equally spaced intervals and using these intervals as categories. The number of intervals is determined as $q = \max \left \{ \min \left \{ \frac{n}{3}, 10 \right \}, 2\right \}$ where $n$ is the number of observations in the data set [55]. Continuous features are categorized analogously. The categorized version of feature $X_k$ is denoted as $X_k^{(c)}$.

Let $X$ and $Y$ be two discrete variables with respective (empirical) probability mass function $p$. Then, the entropy of $Y$ is defined as

(10) \begin{align*}& H(Y) = - \sum_y p(y) \log_2 \left(p(y)\right) \end{align*}

and the conditional entropy of $Y$ given $X$ is given by

(11) \begin{align*} H(Y|X) &= \sum_x p(x) H(Y|X=x) \nonumber \\ &= \sum_x p(x) \left( - \sum_y p(y|x) \log_2 \left(p(y|x)\right)\right). \end{align*}

The entropy measures the uncertainty of a variable. When all possible values occur with roughly the same probability, the entropy is high. If the probabilities of occurrence are very different from each other, the entropy is low. The mutual information of two variables is defined as

(12) \begin{align*}& I(Y; X) = H(Y) - H(Y|X). \end{align*}

It can be interpreted as the decrease in uncertainty about $Y$ conditional on knowing $X$. Considering the symmetry property $I(Y; X) = I(X; Y)$, it can also be seen as the amount of information shared by $X$ and $Y$.

The *mim* filter [36] ranks all features according to the information they share with the target variable $Y^{(c)}$

(13) \begin{align*}& S_{\textrm{mim}}\left(Y^{(c)}, X_k^{(c)}\right) = I\left(Y^{(c)}; X_k^{(c)}\right). \end{align*}

The following filter methods calculate the scores of all features iteratively, implying that the features are selected in a greedy forward manner. Let $G$ denote the set of features that are already selected. $G$ is initialized as $G = \left \{X_k^{(c)}\right \}$ with $k = \underset{j \in \{1, \ldots , p\}}{\textrm{argmax}} \ I\left (Y^{(c)}; X_j^{(c)}\right )$. In each iteration, the feature that maximizes the respective score is added to $G$.

The *mrmr* filter [30] uses the score

(14) \begin{align*}& S_{\textrm{mrmr}}\left(Y^{(c)}, X_k^{(c)}\right) = I\left(Y^{(c)}; X_k^{(c)}\right) - \frac{1}{\left| G \right|} \! \! \sum_{X_j^{(c)} \in G} \! \! \! I\left(X_k^{(c)}; X_j^{(c)}\right). \end{align*}

The term $I\left (Y^{(c)}; X_k^{(c)}\right )$ measures the relevance of the feature based on the information this feature has about $Y^{(c)}$. The term $\frac{1}{\left | G \right |} \sum _{X_j^{(c)} \in G} I\left (X_k^{(c)}; X_j^{(c)}\right )$ judges the redundancy of $X_k^{(c)}$ by assessing the mean information that the feature shares with the features in $G$. The idea is to find maximally relevant and minimally redundant (mrmr) features.

For the *jmi* filter [56], the score

(15) \begin{align*}& S_{\textrm{jmi}}\left(Y^{(c)}, X_k^{(c)}\right) = \sum_{X_j^{(c)} \in G} I\left(Y^{(c)}; X_k^{(c)}, X_j^{(c)}\right) \end{align*}

is employed. $I\left (Y^{(c)}; X_k^{(c)}, X_j^{(c)}\right )$ is the amount of information about $Y^{(c)}$ that $X_k^{(c)}$ and $X_j^{(c)}$ provide jointly. This quantity can be calculated by using the variable $X = \left (X_k^{(c)}, X_j^{(c)}\right )^\top $ and its multivariate probability mass function in the definition of mutual information. The idea of this score is to include features that are complementary to the already selected features.

The *jmim* filter [57] is a modification of the *jmi* filter. The score

(16) \begin{align*}& S_{\textrm{jmim}}\left(Y^{(c)}, X_k^{(c)}\right) = \underset{X_j^{(c)} \in G}{\min}\left\{I\left(Y^{(c)}; X_k^{(c)}, X_j^{(c)}\right)\right\} \end{align*}

considers the minimal joint information over all already selected features instead of the sum.

For the *disr* filter [58], the score

(17) \begin{align*}& S_{\textrm{disr}}\left(Y^{(c)}, X_k^{(c)}\right) = \sum_{X_j^{(c)} \in G} \frac{I\left(Y^{(c)}; X_k^{(c)}, X_j^{(c)}\right)}{H\left(Y^{(c)}, X_k^{(c)}, X_j^{(c)}\right)} \end{align*}

is used. Like the *jmi* filter, it uses the information about $Y^{(c)}$ provided jointly by $X_k^{(c)}$ and $X_j^{(c)}$. But additionally, this information is divided by the joint entropy of $Y^{(c)}$, $X_k^{(c)}$, and $X_j^{(c)}$. To obtain this entropy, consider the variable $Y = \left (Y^{(c)}, X_k^{(c)}, X_j^{(c)}\right )^\top $ and plug it into the above definition of the entropy.

The *njmim* filter [57] is a modification of the *disr* filter. Its score

(18) \begin{align*}& S_{\textrm{njmim}}\left(Y^{(c)}, X_k^{(c)}\right) = \underset{X_j^{(c)} \in G}{\min}\left\{\frac{I\left(Y^{(c)}; X_k^{(c)}, X_j^{(c)}\right)}{H\left(Y^{(c)}, X_k^{(c)}, X_j^{(c)}\right)}\right\} \end{align*}

considers the minimal relative joint information over all already selected features instead of the sum.

The *cmim* filter [59] has the score

(19) \begin{align*}& S_{\textrm{cmim}}\left(Y^{(c)}, X_k^{(c)}\right) = \underset{X_j^{(c)} \in G}{\min}\left\{I\left(Y^{(c)}; X_k^{(c)} | X_j^{(c)}\right)\right\}. \end{align*}

It uses the conditional mutual information

(20) \begin{align*}& I\left(Y^{(c)}; X_k^{(c)} | X_j^{(c)}\right) = H\left(Y^{(c)} | X_j^{(c)}\right) - H\left(Y^{(c)} | X_k^{(c)}, X_j^{(c)}\right) \end{align*}

that can be interpreted as the difference in uncertainty about $Y^{(c)}$ before and after $X_k^{(c)}$ is known, while $X_j^{(c)}$ is known anyway. The idea is to select features that provide much information about the class variable, given the information of the already selected features.

#### 2.2.4 Implementation of filter methods

**Table 1:** Overview of the filter methods: Name of the filter method (filter), short description of filter (description), information if filter is multivariate (multivariate), information if filter uses the survival outcome or a transformed target variable (target), information if filter requires categorization of numeric features (features category), and information about *R* package from which the implementation is taken

Filter	Description	Multivariate	Target	Features category	Implementation
*variance*	Feature variance	No	$T, \Delta $	No	*mlr3filters* [60]
*correlation*	Pearson correlation	No	$Y^{(m)}$	No	*mlr3filters* [60]
*cox.score*	Score test	No	$T, \Delta $	No	*survival* [61]
*carss*	Correlation-adjusted regression survival scores	No	$\log (T), \Delta $	No	*carSurv* [62]
*permutation*	Random forest permutation importance	Yes	$T, \Delta $	No	*ranger* [63] with default hyper parameter settings
*impurity*	Random forest impurity importance	Yes	$T, \Delta $	No	*ranger* [63] with default hyper parameter settings
*boosting*	Boosting importance	Yes	$T, \Delta $	No	*xgboost* [64] with 2 000 boosting iterations, step size 0.05 and maximum tree depth 10
*mim*	Mutual information	No	$Y^{(c)}$	Yes	*praznik* [55]
*mrmr*	Mutual information	Yes	$Y^{(c)}$	Yes	*praznik* [55]
*jmi*	Mutual information	Yes	$Y^{(c)}$	Yes	*praznik* [55]
*jmim*	Mutual information	Yes	$Y^{(c)}$	Yes	*praznik* [55]
*disr*	Mutual information	Yes	$Y^{(c)}$	Yes	*praznik* [55]
*njmim*	Mutual information	Yes	$Y^{(c)}$	Yes	*praznik* [55]
*cmim*	Mutual information	Yes	$Y^{(c)}$	Yes	*praznik* [55]

Table 1 provides an overview of the filter methods and the implementations used for the benchmark experiments in this article.

### 2.3 Feature selection stability measure

Let $V_1, \ldots , V_m$ denote $m$ sets of selected features and $\left | V_i \right |$ the cardinality of set $V_i$. Let $E\left [\cdot \right ]$ denote the expected value for a random selection of two feature sets that have the same cardinality as $V_i$ and $V_j$, respectively (with equal selection probabilities for all sets that have the respective cardinality). Let $\textrm{sim}(X_k, X_l)$ be the similarity of two features $X_k$ and $X_l$, assessed with a similarity measure that attains values in the interval $[0, 1]$, for example the absolute Pearson correlation, and let $\theta \in [0, 1]$ be a threshold. The stability measure SMA-Count [65] is defined as

(21) \begin{align*} &\text{SMA-Count} = \frac{2}{m (m-1)} \sum\limits_{i=1}^{m-1} \sum\limits_{j = i+1}^m S(V_i, V_j) \quad \textrm{with}\\ &S(V_i, V_j) = \frac{\left| V_i \cap V_j \right| \! + \! \textrm{Adj}(V_i, V_j) \! - \! E\left[ \left| V_i \cap V_j \right| \! + \! \textrm{Adj}(V_i, V_j)\right]}{\sqrt{ \left| V_i \right| \!\cdot \! \left| V_j \right|} \! - \! E\left[ \left| V_i \cap V_j \right| \! + \! \textrm{Adj}(V_i, V_j)\right]}, \nonumber \\ &\textrm{Adj}(V_i, V_j) = \min\{ A(V_i, V_j), A(V_j, V_i)\}, \nonumber \\ &A(V_i, V_j) = \left| \{ X_k \! \in \! (V_i \backslash V_j) \!: \exists X_l \! \in \! (V_j \backslash V_i) \!: \textrm{sim}(X_k, X_l) \! \geq \! \theta\} \right|. \nonumber \end{align*}

The term $S(V_i, V_j)$ measures the similarity of the two sets $V_i$ and $V_j$. It takes into account features that are included in both sets ($\left | V_i \cap V_j \right |$) as well as features that are not included in both sets but that are very similar to at least one feature in the other set ($\textrm{Adj}(V_i, V_j)$). The maximum value of SMA-Count is 1 and indicates a perfectly stable feature selection. The stability measure SMA-Count is suitable for data sets that contain highly correlated features such as gene expression data [41].

## 3 Experiments

### 3.1 Data sets and software

For our benchmark experiments, we use 11 high-dimensional survival data sets. All of them contain gene expression data from cancer patients and right-censored survival outcomes. The data sets are taken from [66]. We select data sets that have at least 50 events and we only use the features that provide RNA and miRNA data. The data sets thus do not contain clinical features. An overview of the considered data sets is displayed in Table 2. The *variance* filter is applicable to all of these data sets because the features are measured on the same scale and have not been scaled to unit variance.

**Table 2:** Information about the data sets: number of observations ($n$), number of features ($p$), number of events ($n.e$), and relative number of events ($r.e$)

Data set	$n$	$p$	$n.e$	$r.e$
BLCA	382	23 906	103	0.27
BRCA	735	23 531	72	0.10
HNSC	443	22 313	152	0.34
KIRC	249	23 697	62	0.25
LGG	419	22 942	77	0.18
LUAD	426	24 480	101	0.24
LUSC	418	24 419	132	0.32
OV	219	25 483	109	0.50
PAAD	124	22 960	52	0.42
SKCM	249	23 250	87	0.35
STAD	295	26 814	62	0.21

The benchmark is conducted using the software *R* [67] and the machine learning packages *mlr3* [42], *mlr3filters* [60], *mlr3proba* [68], *mlr3learners* [69] and *mlr3pipelines* [70]. Moreover, the filter implementations are based on the *R* packages *carSurv* [62], *ranger* [63], *xgboost* [64] and *praznik* [55], see Table 1. The *R* package *survival* [61] is employed for fitting unregularized Cox regression models and computing martingale residuals while *glmnet* [71] is used for fitting $L_2$-regularized Cox regression models. The experiments are rolled out on a high-performance compute cluster using the *R* package *batchtools* [72]. The feature selection stability is computed with the *R* package *batchtools* [73]. For analyzing the results, the *R* package *ggplot2* [74] is used. The *R* source code for all analyses presented in this article is publicly available at https://github.com/bommert/survival-filter-benchmark.

### 3.2 Similarity of feature rankings

In this first part of the analyses, the similarity of the filter methods is assessed. The aim is to identify groups of filter methods that rank the features in a similar way.

#### 3.2.1 Experimental setup

The 14 filter methods described in the Methods section are applied to the 11 data sets presented in the previous subsection. Each filter is used to rank all features of each data set. Then, for each data set, the rankings of all filter methods are compared. This is done in the following way: Let $L_i$ and $L_j$ denote the lists of features ordered by the rankings of filters $i$ and $j$, respectively. That is, the first entry of list $L_i$ is the most important feature according to filter method $i$ and the last entry of $L_i$ is the least important feature according to filter method $i$. Let $L_i[1, \ldots , k]$ denote the sublist that contains the first $k$ entries of $L_i$. Then, $\left | L_i[1, \ldots , k] \cap L_j [1, \ldots , k] \right |$ is the number of features that are among the first $k$ features in both lists. Based on theses numbers, the ordered list (OL) score is calculated [75]:

(22) \begin{align*} \textrm{OL}_{ij} &= \sum_{k = 1}^r w_k \cdot \left| L_i[1, \ldots, k] \cap L_j [1, \ldots, k] \right| \quad \textrm{with} \\ w_k &= \frac{r + 1 - k}{\sum_{i = 1}^r i (r + 1 - i)}. \nonumber \end{align*}

This score is a weighted sum of the numbers of features included in both lists, giving more importance to features placed in the first positions. The weights are linearly decreasing with the lengths of the considered sublists and scaled such that the maximum value of the OL score is 1. The parameter $r$ determines the maximal lengths of the sublists. The OL score is taken from [75], but we use linearly decreasing weights instead of exponentially decreasing weights. The reason is that with exponentially decreasing weights, the OL scores are mostly determined by the first few positions of the sublists, giving almost no relevance to the rest of the sublists.

**Figure 1:**
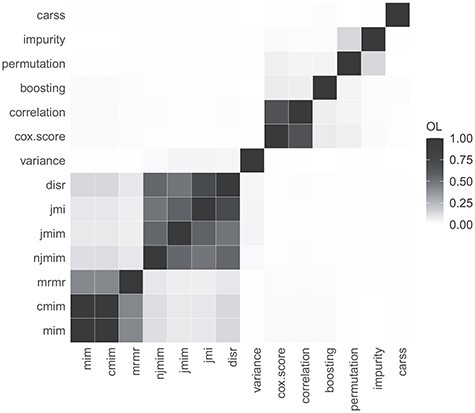
Mean OL scores between the filter methods averaged across data sets. The filter methods are ordered based on single linkage clustering.

#### 3.2.2 Results

In order to assess the similarity of the filter methods, for each data set, OL scores are computed for all pairs of filter methods. Then, the scores are aggregated across data sets with the arithmetic mean. For the computation of the OL scores, only the $r=100$ top ranked features are considered. Figure 1 displays the mean OL scores. Three groups of similar filter methods can be identified. The first group consists of the mutual information filters *mim*, *cmim* and *mrmr*. The second ground is formed by the remaining mutual information filters *njmim*, *jmim*, *jmi* and *disr*. The third group consists of the filters *cox.score* and *correlation*. There exist weak similarities between the two groups of mutual information filters as well as between the two random forest feature importance filters *permutation* and *impurity*. The other filter methods are not similar in the way they rank the top features.

It is plausible that the mutual information filters yield similar feature rankings because they use similar concepts for score calculation and they categorize the features in the same way. Especially for the second group of mutual information filters, the high similarity values make sense because all of these filters rank the features iteratively with respect to joint mutual information terms. Considering the filters *mim* and *cmim*, it is rather surprising that they are so highly similar, because *mim* is a univariate filter method and *cmim* iteratively assesses conditional mutual information terms based on the already selected features. A possible reason for this similarity could be that the features do not provide much information about the target variable, making the conditional mutual information term in $S_{\textrm{cmim}}$ take on similar values as the mutual information term in $S_{\textrm{mim}}$. Regarding the filter methods *cox.score* and *correlation*, their similarity can be explained by both filters being indicators of univariate associations between the features and the target.

### 3.3 Comparison of the performance

#### 3.3.1 Experimental setup

The aim of this analysis is to identify the best filter methods with respect to predictive performance and to run time. To assess the predictive performance of the filter method, each filter method is combined with $L_2$-regularized Cox regression [45] such that the filter is applied first and the Cox model is trained only on the features selected by the filter. We choose $L_2$-regularized Cox regression because it is a frequently used regression technique that is known to perform well [76] and it does not perform embedded feature selection. The latter aspect is important for judging the predictive quality of the entire set of selected features.

**Figure 2:**
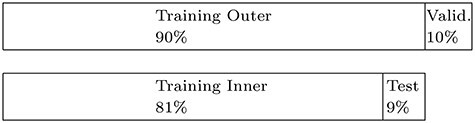
Visualization of nested cross-validation.

Each filter method has one hyper parameter, *prop*, indicating the proportion of features to be selected by the filter. To tune this hyper parameter, we perform a grid search. We consider the 100 equidistant values $\{0.01, 0.02, \ldots , 1\}$ and transform them with $x \mapsto x^2$, focusing more on small proportions of selected features. We conduct nested cross-validation [77] with 10 inner and 10 outer iterations (see Figure 2 for a visualization). Both the inner and the outer cross-validation splits are stratified based on the event indicator $\Delta $. For each filter method and data set, in each outer iteration, we consider the best *prop*-value based on the inner cross-validation. For choosing the best *prop*-value, the filter method ranks the features on the inner training data sets. For each of the 100 *prop*-values, the best $\textit{prop} \cdot 100 \%$ of the features are used to train a $L_2$-regularized Cox regression model on the inner training data sets. Then, the 10 Cox models are evaluated with respect to predictive performance on the respective test data sets. Based on the mean predictive performance, the best of the 100 *prop*-values is chosen. The next step is to evaluate the performance of the filter method on the validation data set. For this, the filter is applied on the outer training data set, selecting $\textit{prop} \cdot 100 \%$ of the features (with *prop* set to the chosen value). Then, a $L_2$-regularized Cox model is fitted on the selected features of the outer training data set. The time for filtering and model fitting on the outer training data set as well as the time for predicting on the validation data set is recorded. Also, the predictive performance on the validation data set is assessed. This procedure is repeated for all 10 outer cross-validation iterations, resulting in 10 values per filter method, data set, and performance criterion.

In addition to the 14 filter methods, we also consider the approach of not applying any filter before fitting the $L_2$-regularized Cox model, which serves as a baseline. For this approach, there exists no hyper parameter *prop* that would require tuning. Therefore the $L_2$-regularized Cox model is directly fitted on the outer training data sets, using all features, and it is evaluated as described above. Moreover, we also include the simple Kaplan–Meier estimator of the survival function [44] as a baseline. The Kaplan–Meier estimator does not consider any of the features.

The hyper parameter of $L_2$-regularized Cox regression that balances the goodness of the fit and the size of the regression parameters is chosen automatically by [71] using 10-fold cross-validation. The predictive performance is evaluated with the integrated Brier score. To ensure a fair comparison of the filter methods, the same inner and outer cross-validation splits are used for all filter methods.

**Figure 3:**
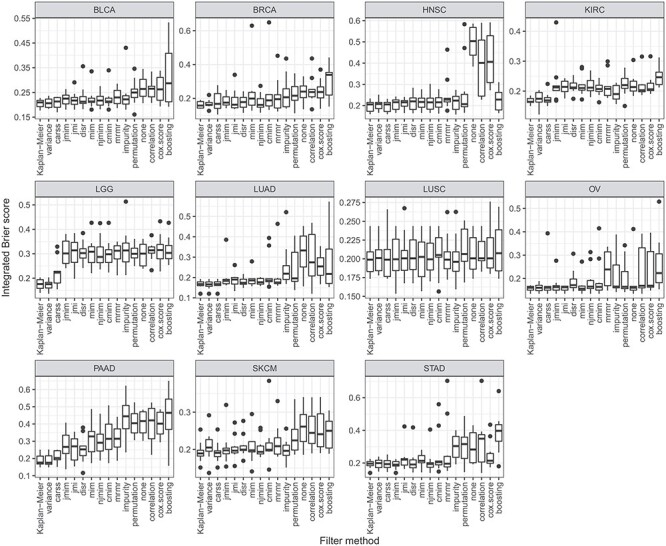
Boxplots of the integrated Brier score of the best configurations of the 10 outer cross-validation iterations per data set. Each boxplot represents 10 performance values. Small values indicate a good predictive performance.

#### 3.3.2 Results

First, the filter methods are compared with respect to predictive performance. Figure 3 shows the integrated Brier score values of the best configurations of the 10 outer cross-validation iterations separately for each data set. It can be observed that for all data sets, there are some filter methods that lead to considerably better results than other filter methods. However, many of the boxplots overlap. For most data sets, applying the filter methods *variance* or *carss* before fitting the $L_2$-regularized Cox model, results in models with high predictive accuracy. Applying no filter before fitting the $L_2$-regularized Cox model leads to comparably bad results on most data sets. The variation of the results between the 10 outer cross-validation iterations is rather small for the filter methods that achieve good results. In comparison to the simple Kaplan–Meier estimator, no gain in predictive performance is achieved with filtering and $L_2$-regularized Cox regression.

**Figure 4:**
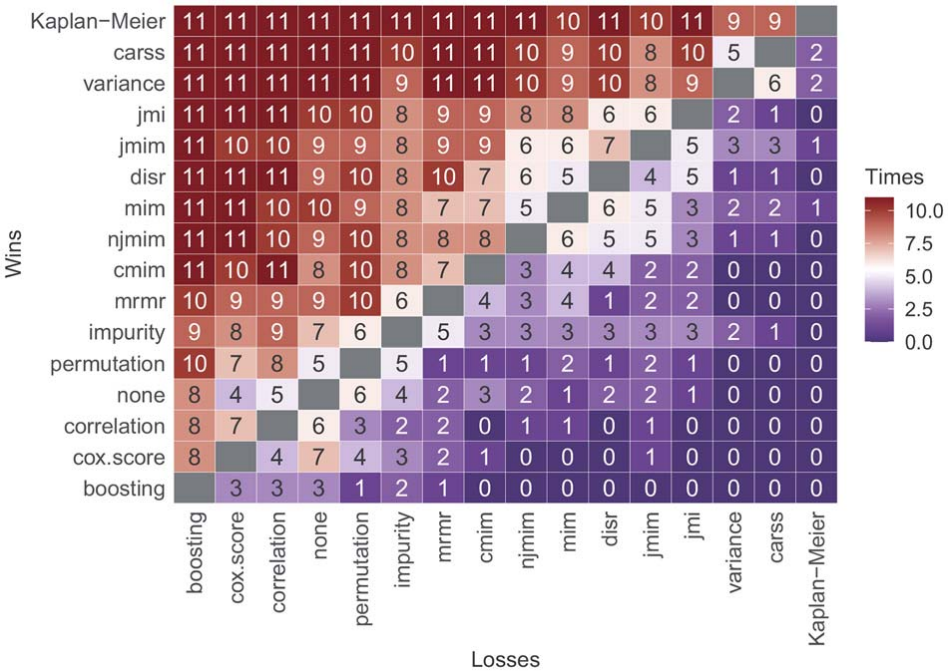
Number of data sets on which the filter method displayed in the row achieves a lower mean integrated Brier score (corresponding to a better performance) than the filter method displayed in the column. The filter methods are ordered by the sums of the rows, that is, the total number of wins against any of the other filter methods on any of the 11 data sets.

Figure 4 provides an aggregation of the predictive performances of the filter methods over all data sets. In order to obtain one performance value per filter method and data set, the mean integrated Brier score of the 10 outer cross-validation iterations is considered. Then, for each pair of filter methods, it is investigated, on how many data sets one filter outperforms the other. In Figure 4, the number displayed in the row of filter *A* and in the column of filter *B* indicates the number of data sets on which filter *A* achieves a lower mean integrated Brier score than filter *B*. Filters *carss* and *variance* achieve lower mean integrated Brier scores than most other filter methods on all data sets. They perform better than *boosting*, *cox.score*, *correlation*, *permutation*, *mrmr* and *cmim* as well as applying no filter on all of the 11 considered data sets. In comparison to the filters *impurity*, *mim*, *njmim*, *disr*, *jmim* and *jmi*, the *carss* and *variance* filters resulted in a better performance on at least 8 of the data sets. Filter *carss* wins against filter *variance* on 5 data sets and loses on 6 data sets. The simple Kaplan–Meier estimator outperforms all other approaches on at least 9 of the 11 data sets, however, compared to the filters *variance* and *carss*, almost always only by a very small margin.

**Figure 5:**
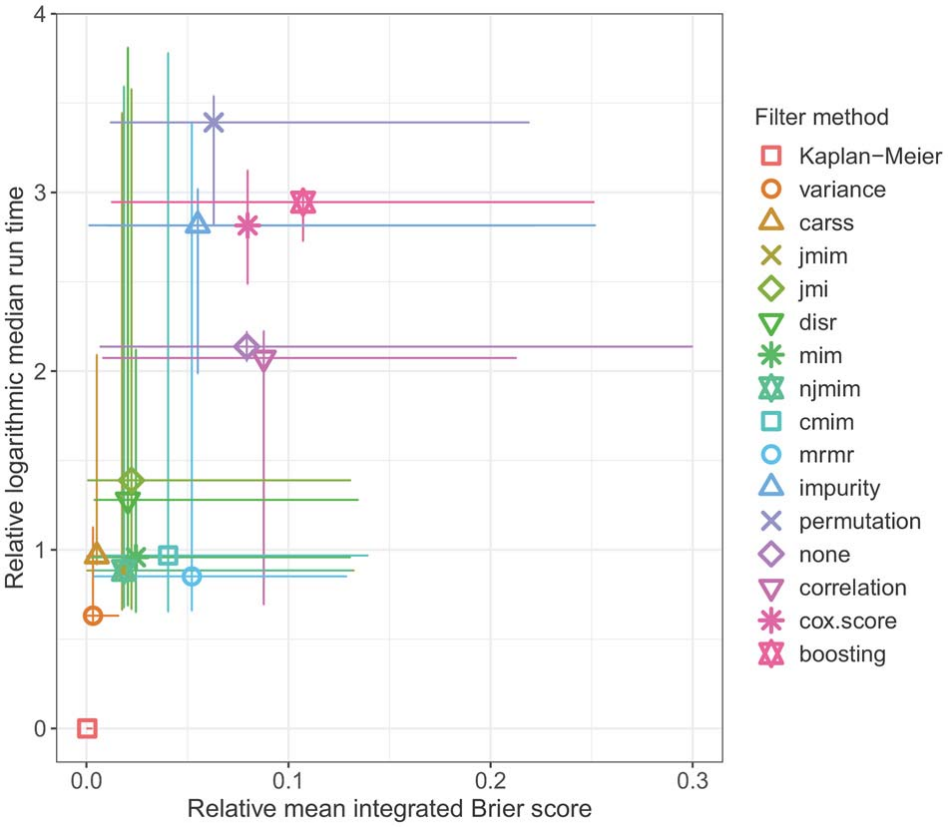
Relative mean integrated Brier score and relative logarithmic median run time. A symbol indicates the median across the 11 data sets. The lines span from the minimum to the maximum value observed for any of the data sets. Small values are desirable for both criteria.

In the next step, we jointly consider the run time and the predictive performance of the filters when combined with a $L_2$-regularized Cox regression model. Ideally, a filter method that provides low prediction errors and is fast to compute is desired. Figure 5 presents an aggregation of both performance criteria over the 11 data sets. The sizes of the considered data sets are different (see Table 2) and also the difficulties of the survival prediction tasks differ between the data sets (see Figure 3). This makes it necessary to scale both performance criteria before they can be aggregated across data sets. Regarding the prediction error, for each data set, we subtract the best observed mean integrated Brier score from all mean integrated Brier scores. The best filter method per data set therefore has ‘relative mean integrated Brier score’ 0. A relative mean integrated Brier score of $x$ means that the predictive performance of a filter method is worse by the additive factor $x$ compared to the best filter method on the same data set. Regarding the run times, we consider the (base 10) logarithmic median run time of the 10 outer cross-validation iterations for each filter and data set. We subtract the fastest logarithmic median run time per data set from all logarithmic median run times measured on the same data set. A ‘relative logarithmic median run time’ of $x$ means that the median run time of a filter equals the median run time for the fastest filter on the same data set multiplied with $10^x$. The scaled performance criteria are displayed in Figure 5 in the following way: The median of both criteria across the 11 data sets is displayed by a symbol. Horizontal and vertical lines reaching from the minimum to the maximum value of the respective performance criterion observed on any of the 11 data sets are added. The symbol represents the central location of the performance measures while the lines indicate the spread across data sets. The Kaplan–Meier estimator requires the least run time among all considered approaches as it does not take into account the features. Also, it provides the highest predictive accuracy on most of the data sets. Among the filter methods, looking at the symbols, it can be seen that filter *variance* outperforms all other filter methods as well as applying no filter. It obtains a better median relative mean integrated Brier score and a better median relative logarithmic median run time than all other filter methods. Filter *carss* also provides a very small median relative mean integrated Brier score but requires more time for calculation. The filters *jmim*, *njmim*, *mim*, *cmim* and *mrmr* are comparably fast to compute but achieve a noticeably lower predictive performance.

**Figure 6:**
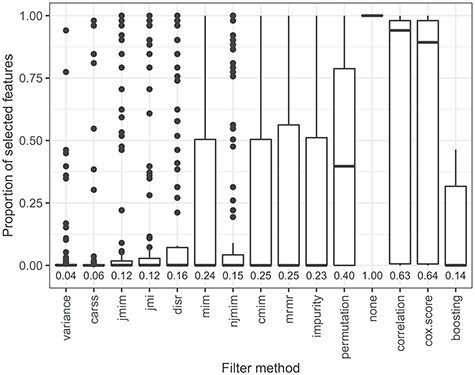
Boxplots of the proportion of selected features of the best configurations of the 10 outer cross-validation iterations for all data sets. Each boxplot represents 110 proportion values. The mean proportions of selected features are indicated by numbers.

In the next step, the feature selection of the filter methods is analyzed in more detail. Figure 6 displays the proportion of selected features per filter method for the best configurations. Remember that for each filter method, the proportion of features to select is optimized based on the predictive performance of the subsequent $L_2$-regularized Cox model. The boxplots in Figure 6 represent the proportions of selected features in the 10 outer cross-validation iterations for each of the 11 data sets. The filter methods are sorted by predictive performance. For the filter methods that lead to a good predictive performance, comparably few features are selected for all data sets. This means that it is sufficient to include only a small number of features in the survival models for obtaining high predictive accuracy. Fitting models based on only a small number of features has the advantage of a faster run time and lower memory consumption as well as an easier interpretation of the $L_2$-regularized Cox model. The comparably bad predictive performance of applying no filter before fitting the $L_2$-regularized Cox model shows that it is not only sufficient but also necessary to filter out many of the features in the considered data sets. The filter methods with low predictive accuracy select comparably many features. This means that they fail at selecting only the few important features for target prediction.

**Figure 7:**
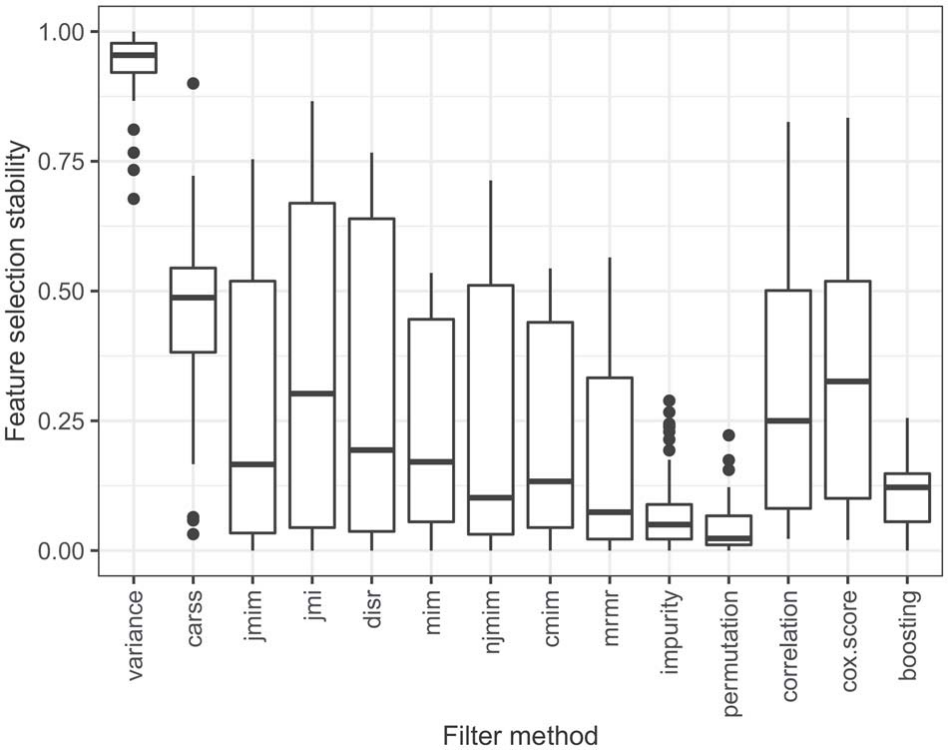
Boxplots of the feature selection stability of the best configurations of the 10 outer cross-validation iterations for all data sets. Each boxplot represents 110 stability values assessed with the stability measure SMA-Count. Large values are desirable.

In Figure 7, the feature selection stability of the filter methods is assessed. For each filter method and each outer iteration of each data set, the following is done: the 10 sets of features that were selected by the filter method during the inner cross-validation iterations with the best *prop*-value are considered. The value of the stability measure SMA-Count (see Equation 21) is calculated, using the absolute Pearson correlation between the features as a measure of feature similarity and the similarity threshold $\theta = 0.9$, which implies a strong association. Large stability values are desirable, as they indicate a consistent choice of features irrespective of some variation in the data set. Figure 7 shows that the feature selection conducted by filter *variance* is by far the most stable among all considered filter methods. The other filter methods provide a much less stable feature selection. Filter *carss* is the second best filter method with respect to feature selection stability.

Finally, we compare the results of the similarity analyses and the performance analyses. The groups of similar filter methods identified on the basis of Figure 1 also provides a similar predictive accuracy. They are positioned next to each other in the ranking in Figure 4 and they obtain similar scaled predictive performance values in Figure 5. Furthermore, the groups of similar filters also select similar proportions of features (see Figure 6) with a similar feature selection stability (see Figure 7) for the respective optimal *prop*-values. All in all, the results of the two analyses are consistent.

## 4 Conclusion

Feature selection is a fundamental problem in statistical research, especially for the analysis of high-dimensional biomedical data sets. It is often advantageous with respect to predictive performance, run time and interpretability to disregard the irrelevant and redundant features. Filter methods are a popular class of feature selection methods because they are fast to compute and can be combined with any subsequent predictive model.

We considered gene expression survival data, which is a typical example of high-dimensional, censored data used in bioinformatics. Existing benchmark studies of filter methods are conducted on the basis of classification or regression data sets. But commonly used regression or classification techniques are not suitable for survival data sets, because they cannot take into account the censoring information. Since censored survival data are frequently used in bioinformatics and since filter methods for survival data had not been thoroughly benchmarked before, we conducted such a benchmark for survival data.

We compared 14 filter methods based on 11 high-dimensional survival data sets containing gene expression data. The data sets were chosen as a subset of the data sets used in [66] based on the number of events. This choice of data sets can be seen as unbiased. First, we analyzed the orders in which the filter methods rank the top 100 features, identifying groups of similar filter methods. Filter methods that were classified as similar often used similar concepts such as mutual information.

Next, we compared the filter methods with respect to predictive performance when combined with a $L_2$-regularized Cox proportional hazards model. We also included the approach of not applying a filter method before fitting the $L_2$-regularized Cox model in our analyses. We could conclude that the filter methods *variance* and *carss* perform best with respect to the integrated Brier score on all considered data sets. Filter *variance* ranks the features based on their variance, independent of the survival outcome, while filter *carss* computes correlation-adjusted regression survival scores. In comparison to fitting $L_2$-reqularized Cox models with all features, models with much better predictive accuracy were obtained when first applying one of these filters. When considering both the predictive performance and the run time for applying the filter, fitting the model and prediction and then aggregating these performance criteria across data sets, filter *variance* outperformed all other approaches.

Next, we analyzed the feature selection of the filter methods. We observed that all filter methods that lead to models with high predictive accuracy only select a small number of features on all data sets. An analysis of the feature selection stability of the filter methods showed that filter *variance* provides by far the most consistent sets of selected features.

Comparing the results of the similarity and the performance analysis, we found them to be in accordance. We observed that the filter methods that were categorized as similar based on their feature rankings also achieved a similar predictive performance and selected a similar number of features with a similar feature selection stability.

Based on our extensive analyses, we recommend using the simple *variance* filter before fitting a Cox regression model on a high-dimensional gene expression survival data set. This filter method allowed fitting models with the best predictive performance, required the least run time for filter score calculation, model fitting and prediction and at the same time produced the most stable results. When a more elaborate filter method is desired, we recommend the *carss* filter. This filter achieved a comparable predictive accuracy, required a bit more run time and ranked second with respect to feature selection stability.

Both the *variance* and the *carss* filter do not have any hyper parameters (other than the proportion of features to select) that require tuning or a robust choice. This is an advantage over filter methods that are based on feature importance values calculated from models like random forest or boosting. In our benchmark study, we did not tune the hyper parameters of random forest or boosting. Tuning them could have led to an increase in predictive accuracy for the respective filter methods. This, however, would have come at the expense of a large increase in run time and the run time of these filters already was the longest among all considered filter methods.

In comparison to the considered mutual information filters, for both the *variance* and the *carss* filter, no categorization of the survival outcome and features, resulting in a loss of information, is required. An advantage of the *variance* filter over the *carss* filter is that it allows unbiased testing in a subsequent unregularized model, if the proportion of features to select is prespecified. This is because the *variance* filter does not take into account the survival outcome for feature selection, see also [78]. A disadvantage of the *variance* filter is that it requires that all features are measured on the same scale, because the feature variance is not scale invariant. On data sets with features that are measured on strongly differing scales, the *variance* filter will likely be misguided. Also, the features must not have been scaled to unit variance during preprocessing. The *carss* filter does not have these limitations.

In conclusion, our results suggest that the filters *variance* and *carss* are a well-performing and convenient tool to eliminate overfitting caused by the high dimensionality of the gene expression data. However, when comparing the integrated Brier score values of the $L_2$-regularized Cox regression models based on the features selected by the best-performing filters to the respective integrated Brier score values of the non-informative Kaplan–Meier estimator, it must be pointed out that the former did not show a clear tendency to outperform the latter. This result is in line with an earlier comparison study by Herrmann *et al*. [66] based on the same data sets, who noted that ‘in general, conclusions about the superiority of one method over the other with respect to the prediction performance must be drawn with caution, as the differences in performance can be very small and the confidence intervals often show a remarkable overlap’ [66]. Furthermore, this finding highlights the importance of the inclusion of clinical (non-genetic) features such as age, sex and disease stage in survival prediction models. Adding clinical covariates to the genetic features could improve the survival predictive performance over the covariate-free Kaplan–Meier estimator. In our study, we only included genetic features in the covariate sets, since these are high-dimensional and subject to filtering, and since we aimed at investigating the performance of filter methods.

Many of the filter methods investigated in this benchmark study are analyzed in [38], too. But there, classification data sets from various domains were used instead of gene expression survival data sets. In [38], especially random forest feature importance filters and mutual information filters performed well with respect to predictive accuracy and run time. The *carss* filter was not analyzed, since it is not suitable for classification data. With the *variance* filter, comparably poor results were obtained. Comparing the findings of this benchmark study with the results of [38], it becomes obvious that the best filter methods differ for different types of data. This stresses the importance of having conducted this benchmark study.

For future analyses one could consider additional data sets from different domains. Also, one could compare the filter methods in simulation studies. In contrast to real data, this would allow assessing whether the filter methods select the features that were used for generating the target. Moreover, one could consider further survival prediction models.

**Key Points**

- On the considered high-dimensional gene expression survival data sets, it is beneficial to first apply a filter method before fitting a $L_2$-regularized Cox proportional hazards model in order to achieve high predictive accuracy.
- The simple variance filter outperforms all other considered filter methods with respect to the predictive accuracy of a subsequent $L_2$-regularized Cox proportional hazards model, the run time and the feature selection stability. This filter selects the features with the largest variance and does not take into account the survival outcome.
- The correlation-adjusted regression scores filter is a more elaborate alternative to the variance filter that allows fitting models with similar predictive accuracy.
- Groups of similar filter methods can be identified based on feature rankings. Filter methods of the same group achieve a similar predictive performance and select a similar number of features with a similar feature selection stability.

## Data Availability

All analyses are reproducible using the R code publicly available on Github. The data sets were derived from OpenML.

## Notes

**Andrea Bommert** is a postdoctoral fellow at the Department of Statistics, TU Dortmund University, Germany.

**Thomas Welchowski** is a postdoctoral fellow at the Institute of Medical Biometry, Informatics and Epidemiology (IMBIE), Medical Faculty, University of Bonn, Germany.

**Matthias Schmid** is a full professor at the Institute of Medical Biometry, Informatics and Epidemiology (IMBIE), Medical Faculty, University of Bonn, Germany.

**Jörg Rahnenführer** is a full professor at the Department of Statistics, TU Dortmund University, Germany.
